# Identification of Suitable Reference Genes for Gene Expression Normalization in the Quantitative Real-Time PCR Analysis of Sweet Osmanthus (*Osmanthus fragrans* Lour.)

**DOI:** 10.1371/journal.pone.0136355

**Published:** 2015-08-24

**Authors:** Chao Zhang, Jianxin Fu, Yiguang Wang, Zhiyi Bao, Hongbo Zhao

**Affiliations:** 1 Department of Ornamental Horticulture, School of Landscape Architecture, Zhejiang Agriculture and Forestry University, Lin’an, Zhejiang, China; 2 Nurturing Station for State Key Laboratory of Subtropical Silviculture, Zhejiang Agriculture and Forestry University, Lin’an, Zhejiang, China; Texas Tech University Health Science Centers, UNITED STATES

## Abstract

Quantitative real-time PCR (RT-qPCR), a sensitive technique for quantifying gene expression, depends on the stability of the reference gene(s) used for data normalization. Several studies examining the selection of reference genes have been performed in ornamental plants but none in sweet osmanthus (*Osmanthus fragrans* Lour.). Based on transcriptomic sequencing data from *O*. *fragrans* buds at four developmental stages, six reference genes (*OfACT*, *OfEF1α*, *OfIDH*, *OfRAN1*, *OfTUB*, and *OfUBC2*) with stable expression (0.5 to 2 fold change in expression levels between any two developmental stages), as well as the commonly used reference gene *Of18S*, were selected as candidates for gene expression normalization in the RT-qPCR analysis of *O*. *fragrans*. For the normalization of RT-qPCR with two dyes, SYBR Green and EvaGreen, the expressional stability of seven candidate reference genes in 43 *O*. *fragrans* samples was analyzed using geNorm, NormFinder and BestKeeper. For RT-qPCR using SYBR Green, *OfRAN1* and *OfUBC2* were the optimal reference genes for all samples and different cultivars, *OfACT* and *OfEF1α* were suitable for different floral developmental stages, and *OfACT* was the optimal reference gene for different temperature treatments. The geometric mean values of the optimal reference gene pairs for the normalization of RT-qPCR are recommended to be used for all samples, different cultivars and different floral developmental stages in *O*. *fragrans*. For RT-qPCR using EvaGreen, *OfUBC2* was the optimal reference gene for all samples and different cultivars, and *OfACT* was the optimal reference gene for different floral developmental stages and different temperature treatments. As the worst reference gene, *Of18S* should not be used as a reference gene in *O*. *fragrans* in the future. Our results provide a reference gene application guideline for *O*. *fragrans* gene expression characterization using RT-qPCR.

## Introduction

As one of ten Chinese traditional flowers, *Osmanthus fragrans* Lour. is particularly appreciated in China for its aesthetic value, unique scent and cultural significance. *O*. *fragrans* cultivars have been divided into 4 groups, Asiaticus, Albus, Luteus, and Aurantiacus [1], according to their ornamental traits (flower color and flowering characteristics). Cultivars in the Asiaticus group flower not just in autumn, whereas cultivars in the other three groups only flower in autumn. These three groups differ substantially in petal color. Cultivars in the Albus group typically have butter-yellow flowers (Royal Horticultural Society Color Chart, RHSCC value of 1 to 8), those in the Luteus group typically show golden yellow flowers (RHSCC value of 9 to 20), and the Aurantiacus Group is characterized by orange/orange-red flowers (RHSCC value of 21 to 30). Previous studies have demonstrated that the petal coloration of *O*. *fragrans* is directly affected by carotenoid composition and content [2,3]. Hence, understanding the expression patterns of carotenoid-related genes in *O*. *fragrans* will help characterize the diverse carotenoid coloration in the flower petals of different *O*. *fragrans* cultivars. Moreover, petal color in this species is sensitive to ambient temperature, but how temperature regulates the petal coloration of *O*. *fragrans* remains unknown. Therefore, investigating the expression of carotenoid-related genes under different temperature conditions will help gain insight into the regulatory mechanisms of environmental factors in *O*. *fragrans* petal coloration.

There are several biological techniques for detecting the expression levels of genes, such as semi-quantitative PCR (semi-PCR), northern blotting, RNase protection assays (RPAs), gene chips, RNA sequencing and quantitative real-time RT-PCR (RT-qPCR). Of these techniques, RT-qPCR is presently regarded as the most reliable method because of its sensitivity, accuracy and high throughput [4–6]. Ideal reference genes (previously known as “housekeeping genes”) are needed as internal controls for normalization in RT-qPCR to quantify the expression level of a target gene. Generally involved in basic cellular processes, traditional reference genes, such as actin (*ACT*), beta-tubulin (*TUB*), elongation factor 1-alpha (*EF1a*), glyceraldehyde-3-phosphate dehydrogenase (*GAPDH*), ubiquitin (*UBQ*) and 18S ribosomal RNA (*18S*) have been widely used as internal controls for gene expression analysis in many plants [7–11]. However, several studies have shown that these traditional reference genes have a smaller variance of expression but are not stably expressed in all experimental conditions, revealing that no reference gene is universally stable [12–14]. Therefore, the identification and validation of potential reference genes in specific experimental conditions is necessary for target gene quantification.

Studies of optimal reference gene selection for gene expression normalization have been conducted in many ornamental plants, such as petunia [15], tree peony [16], *Chrysanthemum lavandulifolium* [17], cineraria [18], rose [19] and *Prunus mume* [10], in addition to model plants and important crops [20–23]. Nevertheless, little information, if any, is available concerning the selection of reference genes in *O*. *fragrans*. To date, *Of18S* is the most widely used reference gene in the semi-PCR and RT-qPCR analyses of *O*. *fragrans* [2,3,8]. However, some studies have demonstrated that the *18S* gene performs poorly as a reference gene for RT-qPCR analyses in *Salvia miltiorrhiza* [24], tree peony [16], Chinese cabbage [11], and watermelon [25]. There is some doubt as to whether *Of18S* is a suitable reference gene in *O*. *fragrans* for gene expression normalization in different cultivars, in different floral development stages or under different temperature treatments. The use of inappropriate reference genes can result in inaccurate measurement of the expression levels of carotenoid-related genes, which may lead to incorrect conclusions with respect to diversified carotenoid coloration in different *O*. *fragrans* cultivars. Thus, the systematic exploration and validation of the most stable reference genes is important and requisite in *O*. *fragrans*.

In this study, in addition to *Of18S*, the commonly used reference gene in *O*. *fragrans*, six candidate reference genes with little variation in expression level in four bud transcriptomes were selected for further study: *ACT*, *EF1α*, NADP-isocitrate dehydrogenase (*IDH*), GTP-binding protein (*RAN1*), *TUB* and Ubiquitin-conjugating enzyme E2 (*UBC2*). We then compared the performance of these seven candidate reference genes in different cultivars, different floral developmental stages and under different temperature treatments using RT-qPCR with two dyes, SYBR Green and EvaGreen. Three algorithms, geNorm [26], NormFinder [27] and BestKeeper [28], were used to determine the most suitable reference gene(s) for the normalization of gene expression in *O*. *fragrans*.

## Materials and Methods

### Plant materials

The *O*. *fragrans* orange-red-flowered cultivar ‘Yanhong Gui’ was used for sample collection from different floral development stages and different temperature treatments. Plants were potted and grown in the resource nursery of Zhejiang Agriculture and Forestry University in Lin’an, Zhejiang Province, China. During bud development (from 9 Aug 2014 to 23 Sep 2014), bud samples of *O*. *fragrans* ‘Yanhong Gui’ were collected weekly, and floral samples (from 23 Sep Aug 2014 to 25 Sep 2014) were collected daily during flower opening. In total, 10 samples during bud development and flower opening constituted the experimental samples of different floral development stages. To collect samples under different temperature treatments, *O*. *fragrans* ‘Yanhong Gui’ plants with globular-shaped buds were treated at 12, 15, 19 and 32°C. To monitor floral development, flower petal samples under four temperature treatments were collected at four developmental stages (linggeng, half opening, full opening and initial senescence), as well as petal samples before the treatments, generating 17 petal samples under different temperature treatments. Additionally, petal samples were collected from the fully opened flowers of 16 cultivars, including ‘Hangzhou Huang’, ‘Jinqiu Gui’ and ‘Yuanban Jingui’ from the Luteus group; ‘Xiaoye Sugui’ and ‘Yu Linglong’ from the Albus group; ‘Chenghong Dangui’, ‘Mantiao Hong’, ‘Wuyi Dangui’, ‘Yanhong Gui’, ‘Yingye Dangui’, ‘Zhusha Dangui’ and ‘Zhuangyuan Hong’ from the Aurantiacus group; and ‘Chenghuang Siji Gui’, ‘Ri Xiang Gui’, ‘Tian Nv San Hua’ and ‘Tian Xiang Tai Ge’ from the Asiaticus group. In total, 43 experimental samples comprised 10 at various stages of floral development, 17 exposed to various temperature treatments, and 16 from different cultivars. All samples were immediately frozen in liquid nitrogen after collection and stored at -80°C until use.

### RNA extraction, quality control and cDNA synthesis

Total RNA from all samples was extracted using the RNAprep Pure Plant Kit (Tiangen, China). All RNA samples were adjusted to the same concentration after measuring the RNA concentration on a NanoDrop ND-1000 spectrophotometer (NanoDrop Technologies). The quality of the RNA was further verified using 1.5% (w/v) agarose gel electrophoresis and ethidium bromide staining. The first strand cDNA was synthesized using 1 μg of total RNA with the Reverse Transcriptase M-MLV (Takara, Japan) according to the manufacturer’s protocol.

### Selection of candidate reference genes

In our preliminary study, four normalized cDNA libraries from *O*. *fragrans* ‘Yanhong Gui’ buds at four developmental stages were constructed and sequenced using the Illumina HiSeq2000 platform (unpublished data). A total of 184,860 unigenes were identified, and the relative expression levels of these unigenes were analyzed at four developmental stages. The level of gene expression was determined by calculating the number of unambiguous tags for each gene and then normalizing this to the number of transcripts per million tags (TPM). The difference in gene expression between the samples was determined using the TPM value. Six reference genes (*OfACT*, *OfEF1α*, *OfIDH*, *OfRAN1*, *OfTUB*, and *OfUBC2*) exhibiting stable expression (0.5–2 fold change in expression level) at four developmental stages (Table 1), as well as *Of18S*, the commonly used reference gene in *O*. *fragrans* [8], were selected as candidates for gene expression normalization in the quantitative real-time PCR analysis of *O*. *fragrans*.

**Table 1:** Expression of the candidate reference genes in the transcriptomic sequencing data.

Gene name	Function	Expression (TPM)				Fold change					
		S1	S2	S3	S4	S2/S1	S3/S2	S3/S1	S4/S3	S4/S2	S4/S1
*OfACT*	Actin	31.897	28.050	32.974	31.683	0.879	1.176	1.034	0.961	1.130	0.993
*OfEF1α*	Elongation factor-1α	99.761	73.916	87.638	74.726	0.741	1.186	0.878	0.853	1.011	0.749
*OfIDH*	NADP-isocitrate dehydrogenase	10.117	8.567	12.444	7.237	0.847	1.453	1.230	0.582	0.845	0.715
*OfRAN1*	GTP-binding protein RAN1	0.051	0.046	0.031	0.047	0.902	0.674	0.608	1.516	1.022	0.922
*OfTUB*	Beta-tubulin	0.984	1.013	1.007	1.029	1.029	0.994	1.023	1.022	1.016	1.046
*OfUBC2*	Ubiquitin-conjugating enzyme E2	1.159	1.534	0.829	1.384	1.324	0.540	0.715	1.669	0.902	1.194

### PCR primer design and test of amplification efficiency

Besides *Of18S*, primers for other six candidate reference genes were designed using Primer Premier 5 with amplicon lengths of 75–143 bp (Table 2). *Of18S* expression analysis was performed with primers that have been widely used previously [2,8]. A gene specificity test for all primer sets was performed using RT-qPCR as previously described [29]. The efficiency of each primer set was evaluated by producing a standard curve using serial dilutions of a cDNA mixture from four developmental buds.

**Table 2:** Reference gene primer sequences and amplicon characteristics using SYBR Green or EvaGreen.

Gene name	Forward primer sequence (5′-3′)	Reverse primer sequence (5′-3′)	Amplicon length (bp)	SYBR Green		EvaGreen	
				PCR efficiency (%)	Regression coefficient (R^2^)	PCR efficiency (%)	Regression coefficient (R^2^)
*OfACT*	CCCAAGGCAAACAGAGAAAAAAT	ACCCCATCACCAGAATCAAGAA	143	109.6	0.9984	102.2	0.9992
*OfEF1α*	CGTTTGCCACTTCAGGATGTCTA	GTACCAGGTTTCAGGACTCCAGTTT	89	97.7	0.9974	101.8	0.9972
*OfIDH*	CTTGAAGCAGATGTGGAAGAGTC	CTTTGTCCATCCTGGGACCAGTC	118	101.8	0.9952	94.6	0.9978
*OfRAN1*	AGAACCGACAGGTGAAGGCAA	TGGCAAGGTACAGAAAGGGCT	117	100.4	0.9903	94.5	1.0000
*OfTUB*	AGAAGGGATGGATGGAATGGA	GTCTTCTTCGTCCTCGGCAGT	106	103.8	0.9981	97.8	0.9955
*OfUBC2*	TGTTGACAAAACCGATGGAAGGA	GTGGAGTGTGGAGGATAAGGGTG	75	97.7	0.9948	92.8	0.9969
*Of18S*	AGCCTGAGAAACGGCTACCAC	ATACGCTATTGGAGCTGGAA	208	104.7	0.9926	106.0	1.0000

### RT-qPCR

RT-qPCR was performed on an ABI 7300 real-time PCR instrument (AppliedBiosystems, Foster City, CA) using SYBR Green or EvaGreen dyes to detect dsDNA synthesis. For the RT-qPCR with SYBR Green, the reaction mixture (20 μL total volume) contained 10 μL of SYBR Premix Ex Taq II (TaKaRa, Japan), 0.8 μL of each primer (10 μM), 2 μL of diluted cDNA (~ 50 ng), 0.4 μL of 50× ROX Reference Dye and 6 μL of ddH_2_O. The PCR program was performed as follows: an initial denaturation at 95°C for 30 s, 40 cycles of 95°C for 5 s and 60°C for 31 s, and a melting curve analysis with a temperature ramp from 60°C to 95°C. For the RT-qPCR with EvaGreen, the reaction mixture (20 μL total volume) contained 10 μL of 2× HRM Analysis PreMix (with EvaGreen) (Tiangen, China), 0.6 μL of each primer (10 μM), 2 μL of diluted cDNA (~ 50 ng), 0.4 μL of 50× ROX Reference Dye and 6.4 μL of ddH_2_O. The PCR program was performed as follows: an initial denaturation at 95°C for 2 min, 40 cycles of 95°C for 5 s and 60°C for 30 s, and a melting curve analysis with a temperature ramp from 60°C to 95°C. No-template controls for each primer set were included in every reaction, and the real-time RT-PCR was performed in triplicate.

### Reference gene expressional stability determination

The expression levels of the seven tested reference genes in all samples were determined using cycle threshold values (Ct). geNorm (version 3.5), NormFinder (version 0.953) and BestKeeper (version 1) [26–28] were used to analyze the expressional stability of the seven candidate reference genes in *O*. *fragrans*.

The geNorm software is a visual basic application (VBA) for determining the most stable reference genes from a set of tested genes by gene expressional stability measure (M). Stepwise exclusion of the gene with the highest M value allows the ranking of the tested genes according to their expression stabilities. Additionally, the pairwise variation (PV) between the sequential normalization factors was calculated to determine the optimal number of reference genes [26]. The NormFinder software used an ANOVA-based model to estimate intra- and inter-group variation and ranked the reference genes according to the stability of their expression patterns in a given sample set under certain experimental conditions [27]. BestKeeper was used to perform numerous pairwise correlation analyses using raw Ct values of each gene and assess reference gene expressional stability using the standard deviation (SD) and the coefficient of variance (CV) of the Ct values [28].

## Results

### Performance of the primers for each reference gene

Six reference genes, *OfACT*, *OfEF1α*, *OfIDH*, *OfRAN1*, *OfTUB*, and *OfUBC2*, were selected as candidate reference genes because they exhibited stable expression (0.5–2 fold change in expression level) during *O*. *fragrans* bud development (Table 1). In addition, the commonly used *O*. *fragrans* reference gene, *Of18S* was also selected as a candidate for gene expression normalization in the quantitative real-time PCR analysis of *O*. *fragrans*. A melting curve analysis of each primer set was performed using RT-qPCR after 40 cycles of amplification. The presence of a single peak indicated that the expected amplicons were amplified with SYBR Green and EvaGreen. The results of the agarose gel electrophoresis demonstrated that all seven primer pairs amplified a single band of the expected size from various cDNA templates. Using SYBR Green, the correlation coefficients (R^2^) ranged from 0.9903 to 0.9984, and PCR amplification efficiencies between 97.7 and 109.6% were obtained from the standard curves generated using a ten-fold serial dilution of cDNA. Using EvaGreen, R^2^ ranged in value from 0.9955 to 1.0000, and PCR amplification efficiencies ranged from 92.8 to 106.0%. These results indicated that each primer set was suitable for gene expression analysis with RT-qPCR using either SYBR Green or EvaGreen.

### Reference gene expression levels

**Fig 1:**
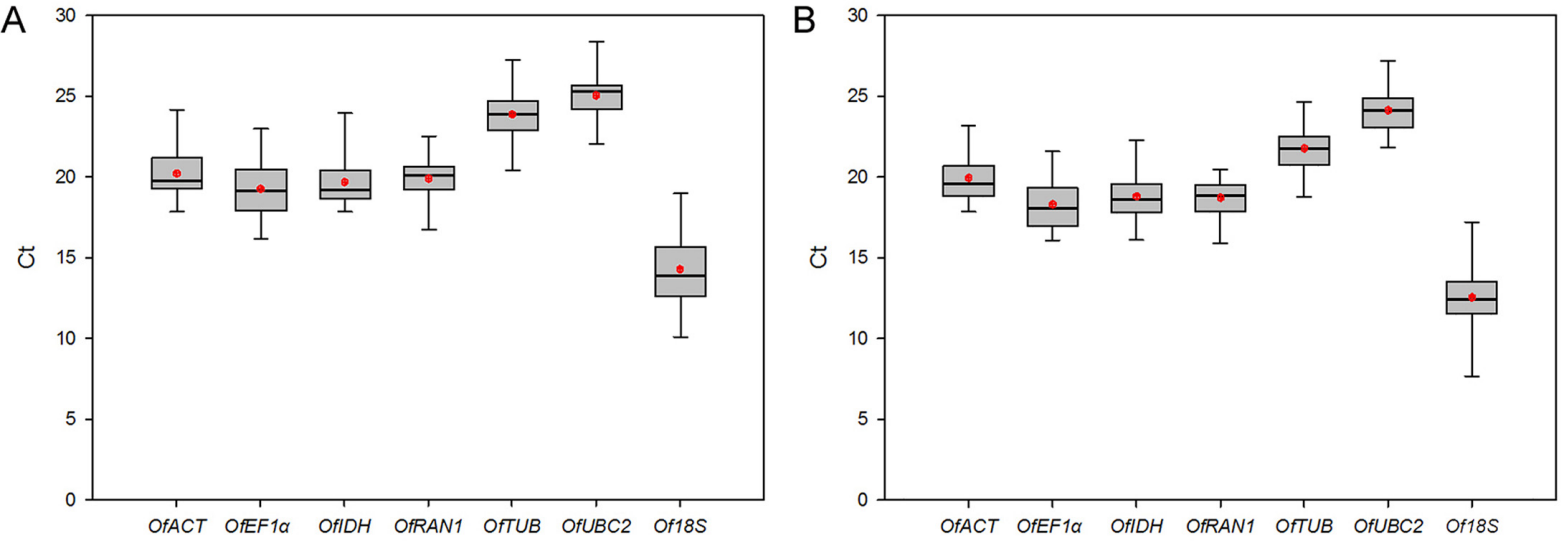
Expression profiles of seven candidate reference genes from 43 samples using SYBR Green (A) or EvaGreen (B). The expression data are displayed as Ct values for each reference gene in all samples. The red point is the mean, and the line across the box is the median. The boxes indicate the 25/75 percentiles. The whisker caps indicate the minimum and maximum values.

The seven candidate reference genes exhibited relatively wide ranges of Ct values using SYBR Green, from 10.09 to 28.37 in 43 tested sample pools, and the mean values of the seven genes were between 14.27 and 25.05 (Fig 1A). The mean values of these seven genes using EvaGreen were between 12.52 and 24.10 (Fig 1), which were generally lower than those using SYBR Green. Using either SYBR Green or EvaGreen, the least abundant transcripts were *OfUBC2* and *OfTUB* with the highest mean Ct values, whereas *Of18S* exhibited the highest expression level with the lowest Cq value of all the samples. In addition, each candidate gene exhibited a specific Ct value variation tendency under the applied conditions. Using SYBR Green, *OfRAN1* exhibited stable gene expression (below 6 cycles), whereas *Of18S* had obvious expression variation (above 8 cycles) as shown in Fig 1A. Similarly, *OfRAN1* also exhibited stable gene expression (below 5 cycles), whereas *Of18S* had obvious expression variation (above 9 cycles) using EvaGreen (Fig 1B).

### geNorm analysis

**Fig 2:**
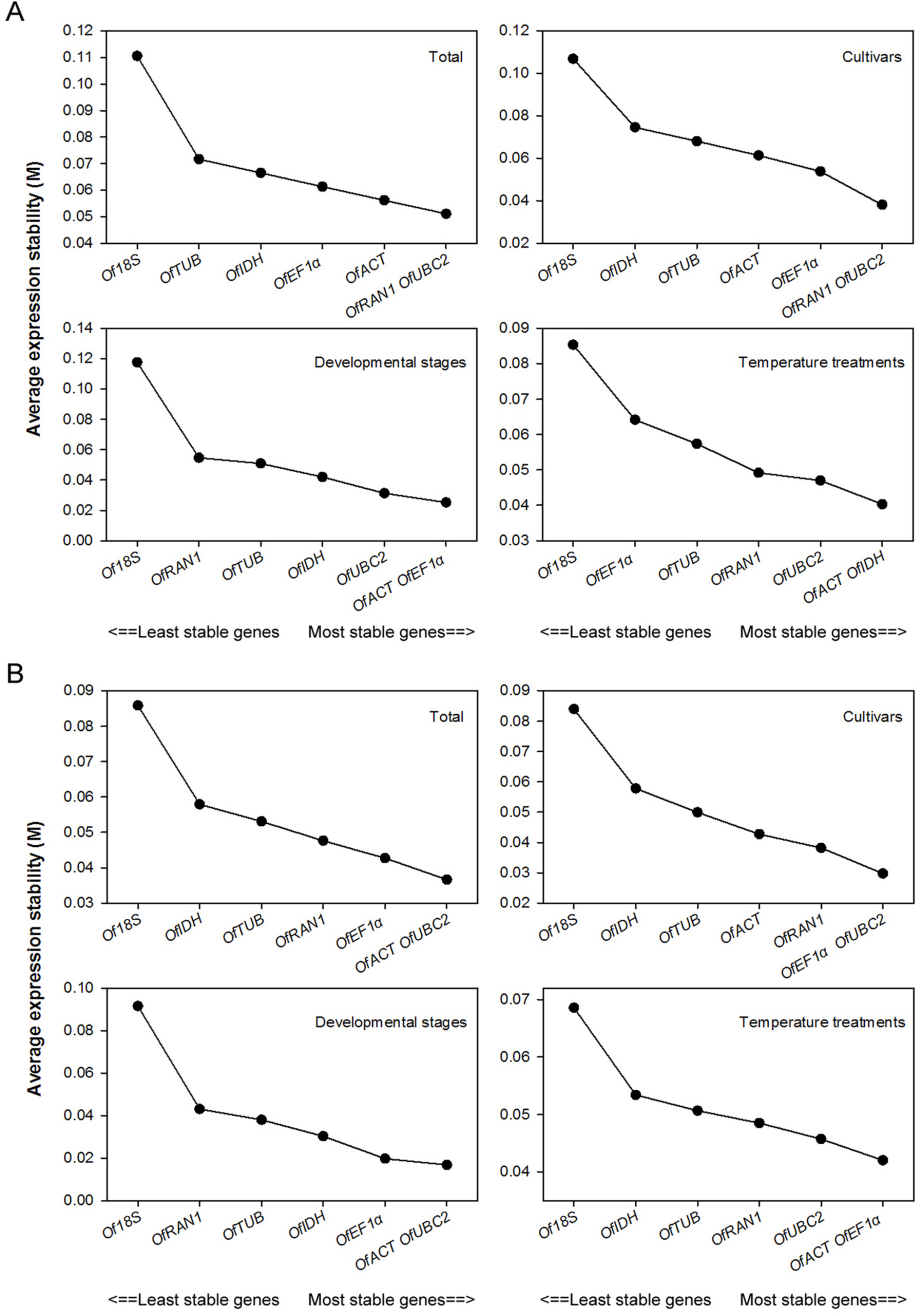
Expressional stability values (M) of seven candidate reference genes in four sample sets using SYBR Green (A) or EvaGreen (B) generated by the geNorm software. Average expressional stability values (M) following stepwise exclusion of the least stable gene across all experimental sets. The least stable genes are on the left, and the most stable genes are on the right.

Before analyzing gene expressional stability, 43 samples were divided into four sample sets: cultivars (16 samples), developmental stages (10 samples), temperature treatments (17 samples), and total (43 samples). We first used geNorm to analyze the expressional stability of the seven candidate reference genes in all the samples and ranked them according to the gene stability index (M): the genes with the lowest M values have the most stable expression (Fig 2). An M value below a threshold of 1.5 is recommended to identify reference genes with stable expression [26]. In this study, the M values of all the reference genes in the four sample sets were much lower than 1.5 (Fig 2).

For all 43 samples, *OfRAN1* and *OfUBC2* were the most stably expressed genes with the lowest M value of 0.051 using SYBR Green (Fig 2A); *OfACT* and *OfUBC2* were the most stably expressed genes with the lowest M value using EvaGreen (Fig 2B). Similarly, *OfRAN1* and *OfUBC2* were also the most stably expressed genes in different cultivars using SYBR Green (Fig 2A). Using EvaGreen, *OfEF1α* and *OfUBC2* were the most stably expressed genes in different cultivars (Fig 2B). For different floral developmental stages, *OfACT* and *OfEF1α* were the most stable with an M value of 0.025 using SYBR Green, whereas *OfACT* and *OfUBC2* were the most stable using EvaGreen (Fig 2). For different temperature treatments, *OfACT* and *OfIDH* were the most stable reference genes using SYBR Green, and *OfACT* and *OfEF1α* were the most stable using EvaGreen (Fig 2). Using either SYBR Green or EvaGreen, *Of18S* was the least stable gene with the highest M value in all four sample sets (Fig 2).

**Fig 3:**
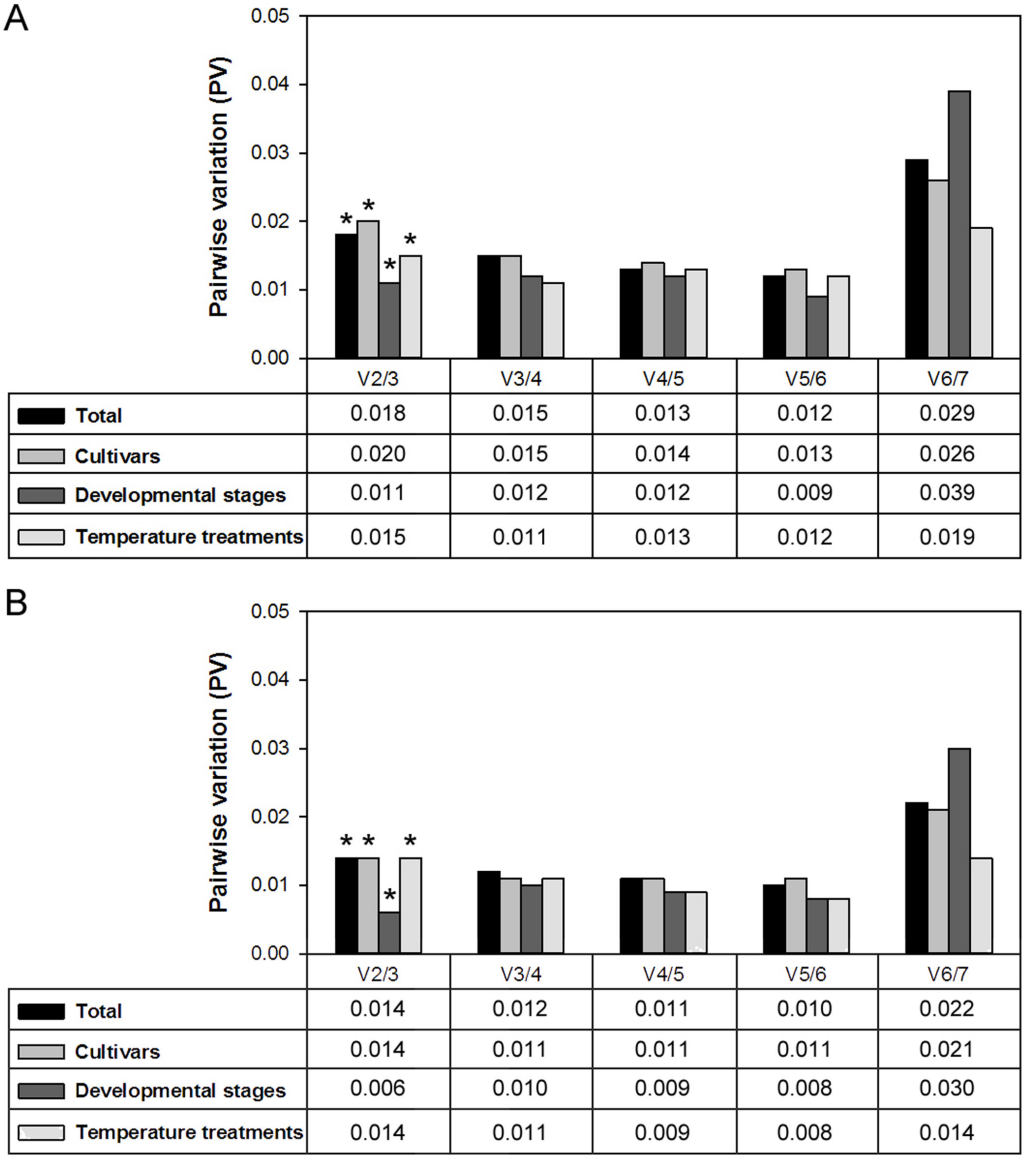
Pairwise variation (PV) analysis of seven candidate genes in four sample sets using SYBR Green (A) or EvaGreen (B). Asterisk indicates the optimal number of reference genes for four sample sets.

The pairwise variation (PV) was also calculated to determine the optimal number of genes required for normalization. The V_n_/V_n+1_ value was lower than 0.15 for all four sample sets using either SYBR Green or EvaGreen (Fig 3), indicating that adding an extra gene to obtain a reliable normalization factor was not necessary in our study. Therefore, two reference genes were necessary and sufficient for gene expression normalization in all sets of samples; i.e., using SYBR Green, the combination of *OfRAN1* and *OfUBC2* was appropriate for all samples and different cultivars, the combination of *OfACT* and *OfEF1α* was appropriate for different floral developmental stages, and the combination of *OfACT* and *OfIDH* was appropriate for different temperature treatments. Using EvaGreen, the combination of *OfACT* and *OfUBC2* was appropriate for all samples and different floral developmental stages, the combination of *OfEF1α* and *OfUBC2* was appropriate for different cultivars, and the combination of *OfACT* and *OfEF1α*was appropriate for different temperature treatments.

### NormFinder analysis

Expression stability was then re-analyzed using the program NormFinder, which is based on a variance estimation approach [27] and ranks the genes according to their stability under a given set of experimental conditions. Using SYBR Green, the ranking generated by this approach was similar to that determined by geNorm because the four most stable genes and the three least stable genes in all sample sets ranked by NormFinder (Table 3) were the same as those generated by geNorm (Fig 2A). According to the results ranked by NormFinder, *OfUBC2* was the most stable gene for all samples and different cultivars (Table 3). *OfACT* and *OfEF1α* were still the most stable genes for different floral developmental stages, whereas *OfACT* was ranked the highest for different temperature treatments using SYBR Green (Table 3). Generally, the rankings using EvaGreen were similar to those using SYBR Green (Table 3). Using EvaGreen, *OfUBC2* was the most stable gene for all samples and different cultivars, and *OfACT* was the most stable gene for different floral developmental stages and different temperature treatments. In addition, using either SYBR Green or EvaGreen, *Of18S* was ranked the lowest in all sample sets (Table 3) by NormFinder, which was in agreement with the ranking of *Of18S* calculated by geNorm.

**Table 3:** Expressional stability analysis of seven candidate reference genes using NormFinder in four sample sets with SYBR Green or EvaGreen.

Rank	Total		Cultivars		Developmental stages		Temperature treatments	
	Gene name	Stability value	Gene name	Stability value	Gene name	Stability value	Gene name	Stability value
SYBR Green								
1	*OfUBC2*	0.010	*OfUBC2*	0.013	*OfACT*、*OfEF1α*	0.009	*OfACT*	0.014
2	*OfACT*	0.013	*OfEF1α*	0.023	*OfUBC2*	0.011	*OfUBC2*	0.020
3	*OfRAN1*	0.016	*OfRAN1*	0.029	*OfIDH*	0.040	*OfRAN1*	0.023
4	*OfEF1α*	0.025	*OfACT*	0.045	*OfRAN1*	0.043	*OfIDH*	0.032
5	*OfIDH*	0.031	*OfTUB*	0.047	*OfTUB*	0.052	*OfEF1α*	0.043
6	*OfTUB*	0.036	*OfIDH*	0.056	*Of18S*	0.189	*OfTUB*	0.047
7	*Of18S*	0.059	*Of18S*	0.125	——	——	*Of18S*	0.090
EvaGreen								
1	*OfUBC2*	0.013	*OfUBC2*	0.007	*OfACT*	0.008	*OfACT*	0.016
2	*OfACT*	0.016	*OfEF1α*	0.018	*OfUBC2*	0.008	*OfRAN1*	0.024
3	*OfEF1α*	0.022	*OfACT*	0.026	*OfEF1α*	0.009	*OfUBC2*	0.025
4	*OfRAN1*	0.029	*OfRAN1*	0.031	*OfIDH*	0.049	*OfEF1α*	0.026
5	*OfTUB*	0.038	*OfTUB*	0.036	*OfTUB*	0.050	*OfTUB*	0.030
6	*OfIDH*	0.043	*OfIDH*	0.044	*OfRAN1*	0.056	*OfIDH*	0.035
7	*Of18S*	0.104	*Of18S*	0.099	*Of18S*	0.230	*Of18S*	0.069

### BestKeeper analysis

BestKeeper, another popular analysis method, was also applied for reference gene expression analysis in this study. Genes with an SD greater than 1 are considered inconsistent; reference genes exhibiting the lowest SD are the most stable genes. Therefore, *OfRAN1* was the most stable gene for all samples and different cultivars using SYBR Green (Table 4), which is in agreement with the results ranked by geNorm but differs from those determined by NormFinder. Using EvaGreen, the rankings generated by BestKeeper were similar to those using SYBR Green for all samples and different cultivars (Table 4). However, for different floral developmental stages and different temperature treatments, the rankings using EvaGreen differed greatly from those using SYBR Green (Table 4). Using SYBR Green, *OfIDH* was the most stable gene for different floral developmental stages, whereas *OfRAN1* was ranked the highest for different temperature treatments (Table 4). However, using EvaGreen, *OfEF1α* was ranked the highest for different floral developmental stages, and *OfTUB* was the most stable gene for different temperature treatments (Table 4). For different floral developmental stages and different temperature treatments using either SYBR Green or EvaGreen, the rankings generated by BestKeeper were quite different from the results determined by geNorm and NormFinder.

**Table 4:** Expressional stability analysis of seven candidate reference genes using BestKeeper in four sample sets with SYBR Green or EvaGreen.

Rank	Total		Cultivars		Developmental stages		Temperature treatments	
	Gene name	Stability value	Gene name	Stability value	Gene name	Stability value	Gene name	Stability value
SYBR Green								
1	*OfRAN1*	0.906	*OfRAN1*	0.548	*OfIDH*	0.383	*OfRAN1*	0.594
2	*OfTUB*	1.100	*OfUBC2*	0.931	*OfACT*	0.536	*OfUBC2*	0.648
3	*OfUBC2*	1.122	*OfTUB*	1.011	*OfEF1α*	0.550	*OfACT*	0.805
4	*OfIDH*	1.125	*OfEF1α*	1.121	*OfUBC2*	0.654	*OfIDH*	0.888
5	*OfACT*	1.176	*Of18S*	1.394	*OfRAN1*	0.857	*OfTUB*	0.914
6	*OfEF1α*	1.396	*OfACT*	1.418	*OfTUB*	0.872	*Of18S*	0.972
7	*Of18S*	1.766	*OfIDH*	1.563	*Of18S*	2.071	*OfEF1α*	1.225
EvaGreen								
1	*OfRAN1*	0.86	*OfRAN1*	0.59	*OfEF1α*	0.21	*OfTUB*	0.67
2	*OfTUB*	1.00	*OfUBC2*	0.99	*OfACT*	0.34	*OfUBC2*	0.72
3	*OfUBC2*	1.03	*OfEF1α*	1.02	*OfIDH*	0.43	*OfRAN1*	0.79
4	*OfACT*	1.08	*OfTUB*	1.19	*OfUBC2*	0.48	*OfACT*	0.83
5	*OfIDH*	1.21	*OfACT*	1.28	*OfTUB*	0.56	*Of18S*	0.93
6	*OfEF1α*	1.21	*Of18S*	1.48	*OfRAN1*	0.64	*OfEF1α*	1.04
7	*Of18S*	1.50	*OfIDH*	1.66	*Of18S*	2.03	*OfIDH*	1.14

With respect to the worst reference gene, *Of18S* was ranked the lowest for all samples and different floral developmental stages using either SYBR Green or EvaGreen (Table 4), which was consistent with the rankings calculated by geNorm and NormFinder. However, BestKeeper indicated that *Of18S* was not the worst reference gene for different cultivars and different temperature treatments, which differs from the results calculated by geNorm and NormFinder (Table 4).

### Overall analysis of the optimal and worst reference genes

The different software tools used to analyze the gene expressional stability in our study, generated different results and different statistical stability values for each gene. The inconsistencies between these three methods were expected because they are based on distinct statistical algorithms. Therefore, the optimal and worst reference genes for each sample set were generated with the aggregated results calculated by geNorm, NormFinder and BestKeeper in our study (Table 5). Using SYBR Green, *OfRAN1* and *OfUBC2* were the optimal reference genes for all samples and different cultivars, *OfACT* and *OfEF1α* were the optimal reference genes for different floral developmental stages, and *OfACT* was the optimal reference gene for different temperature treatments (Table 5). To reduce variation and improve normalization [26], the geometric mean of the optimal reference gene pairs is recommended to be used for the gene expression normalization of RT-qPCR using SYBR Green for all samples, different cultivars and different floral developmental stages in *O*. *fragrans*. Using EvaGreen, *OfUBC2* was the optimal reference gene for all samples and different cultivars, and *OfACT* was the optimal reference gene for different floral developmental stages and different temperature treatments (Table 5). In addition, *Of18S* was the worst reference gene for each sample set using either SYBR Green or EvaGreen (Table 5).

**Table 5:** Optimal and worst reference genes in four sample sets using three methods.

Sample sets	Optimal reference gene				Worst reference gene			
	geNorm	NormFinder	BestKeeper	Aggregated result	geNorm	NormFinder	BestKeeper	Aggregated result
SYBR Green								
Total	*OfRAN1*, *OfUBC2*	*OfUBC2*	*OfRAN1*	*OfRAN1*, *OfUBC2*	*Of18S*	*Of18S*	*Of18S*	*Of18S*
Cultivars	*OfRAN1*, *OfUBC2*	*OfUBC2*	*OfRAN1*	*OfRAN1*, *OfUBC2*	*Of18S*	*Of18S*	*OfIDH*	*Of18S*
Developmental stages	*OfACT*, *OfEF1α*	*OfACT*, *OfEF1α*	*OfIDH*	*OfACT*, *OfEF1α*	*Of18S*	*Of18S*	*Of18S*	*Of18S*
Temperature treatments	*OfACT*, *OfIDH*	*OfACT*	*OfRAN1*	*OfACT*	*Of18S*	*Of18S*	*OfEF1α*	*Of18S*
EvaGreen								
Total	*OfACT*, *OfUBC2*	*OfUBC2*	*OfRAN1*	*OfUBC2*	*Of18S*	*Of18S*	*Of18S*	*Of18S*
Cultivars	*OfEF1α*, *OfUBC2*	*OfUBC2*	*OfRAN1*	*OfUBC2*	*Of18S*	*Of18S*	*OfIDH*	*Of18S*
Developmental stages	*OfACT*, *OfUBC2*	*OfACT*	*OfEF1α*	*OfACT*	*Of18S*	*Of18S*	*Of18S*	*Of18S*
Temperature treatments	*OfACT*, *OfEF1α*	*OfACT*	*OfTUB*	*OfACT*	*Of18S*	*Of18S*	*OfIDH*	*Of18S*

## Discussion

RT-qPCR has emerged as a powerful tool for gene expression analysis, particularly with respect to sensitivity and specificity [30]. Regardless of experimental conditions, the quantitative accuracy of RT-qPCR strongly depends on stably expressed reference genes. However, no one gene has a constant expression profile under all developmental or experimental conditions [30]. A systematic verification of the most suitable reference genes for specific experimental conditions is extremely important for gene expression studies using RT-qPCR in *O*. *fragrans*. Our study compared and analyzed the stability of seven candidate reference genes in four experimental sets, which is the first systematic study of reference gene expressional stability in *O*. *fragrans*. Out of the wide variety of commercially available fluorescent DNA dyes, SYBR Green remains the most widely used DNA dye for RT-qPCR applications despite numerous studies demonstrating that it inhibits PCR in a concentration-dependent manner and affects the DNA melting temperature [31–33]. The EvaGreen dye is marketed as a desirable alternative to SYBR Green because EvaGreen is less inhibitory to PCR and produces sharper peaks in melt curve analyses than SYBR Green [34,35]. Although the gene stability rankings generated by each method were not identical, the four most stable genes and the three least stable genes analyzed using geNorm, NormFinder and BestKeeper were identical in all sample sets. According to the aggregated results in our study (Table 5), the optimal reference gene for RT-qPCR using EvaGreen in each sample set was consistent with that for using SYBR Green because *OfACT* was the optimal reference gene for different temperature treatments using either SYBR Green or EvaGreen, and the optimal reference gene using EvaGreen for the other sample sets was one of the optimal reference genes using SYBR Green, suggesting that the usage of different dyes does not greatly affect the validation of the most stable reference gene.

RAN, an evolutionarily conserved small G-protein family protein, is essential for nuclear transport, nuclear assembly, mRNA processing, and cell cycle control [36–38]. *RAN3*, a homologue of the *RAN* gene from *Antirrhinum majus*, is commonly used as the reference gene for RT-qPCR in this species [39,40]. In our study, *OfRAN1* was recommended as a suitable reference gene for all samples and different cultivars using SYBR Green, suggesting that *OfRAN1* may be used as a reference gene for the normalization of target genes with RT-qPCR using SYBR Green among different *O*. *fragrans* cultivars and in complicated experimental sets. However, *OfRAN1* performed poorly during floral development in *O*. *fragrans* because, using either SYBR Green or EvaGreen, the expressional stability of this gene was close to the bottom of the ranking order generated by the three statistical methods. In petunia, as validated using qBasePlus and geNorm, *RAN1* was regarded as one of the most stably expressed genes during flower development [15], which was not consistent with the results in our study. The inconsistent expressional stability of *RAN1* is probably related to different sampling. In addition to flower opening, samples during bud development were also included in our study; however, only samples from the four flower-opening stages were included in the petunia study [15].

*OfUBC2* was a suitable reference gene for all samples and different cultivars using either SYBR Green or EvaGreen in this study. *OfUBC2* was also stably expressed in different floral developmental stages and different temperature treatments, as shown by the geNorm, NormFinder and BestKeeper analyses. *OfUBC2* is a homologue of the *UBC* gene, a classical traditional reference gene. *UBC* genes from other species also show stable expression in most experimental sets. For example, in hybrid roses, *UBC* was stably expressed in a whole dataset and in different tissues [41]. Moreover, in *Platycladus orientalis*, *UBC* was also top-ranked in all developmental stages and under all stress conditions [42]. In *Arabidopsis thaliana*, the *UBC* gene (at5g25760), is widely employed as the internal control for the normalization of target gene expression under cold treatment [43,44], and this gene has been validated as the only traditional reference gene out of 14 suitable *Arabidopsis* reference genes [44]. Interestingly, this gene was also stably expressed in the set tested and validated by Czechowski et al. [20]. However, *UBC* (at5g25760) is not always stably expressed in *Arabidopsis*; the expressional stability of this gene was low in *Arabidopsis* exposed to cadmium and copper treatments [45].

The traditional reference gene *ACT* is involved in basic cellular processes and has always been considered a potential reference gene in numerous species. In our study, *OfACT* was the optimal reference gene for different floral developmental stages. In many species, *ACT* has also been demonstrated to be stably expressed during the development of specific tissues. For example, *ACT* was stably expressed in the developmental series of soybean [46], during the flower development of petunia ‘V30’ [15], in different fruit developmental stages of *Litchi chinensis* [47] and in the different flower developmental stages of four different color lines in cineraria [18]. Moreover, *ACT* was also stably expressed under various abiotic stresses, including different temperatures, different hormones and wounding [19,42,48]. As in the present study, *OfACT* was also the optimal reference gene for different temperature treatments in *O*. *fragrans*.

For different floral developmental stages, *OfEF-1a* was the other optimal reference gene using SYBR Green and also performed well with EvaGreen. *EF-1a* is regarded as one of the most stable genes in the different fruit developmental stages of *L*. *chinensis* [47]. Moreover, *EF-1a* is also the most stably expressed gene in the six fruit developmental stages of *Litsea cubeba* [49] and in the whole dataset of the flower and leaf development of petunia ‘Mitchell’ [15].

The widely used reference gene *Of18S* had the most obvious expression variation in Ct values in the 43 tested samples (Fig 1). As expected, *Of18S* was ranked the last and regarded as the worst reference gene in all sample sets using either SYBR Green or EvaGreen. Moreover, *Of18S* exhibited a significantly higher expression level with the lowest Cq value of all genes tested, suggesting that this gene is unsuitable for the normalization of target genes with middle or low expression levels. Similarly, in *Salvia miltiorrhiza* [24], tree peony [16], Chinese cabbage [11], and watermelon [25], the *18S* gene was also found to perform poorly as a reference gene.

## Conclusions

In this study, we investigated the expressional stability of seven candidate reference genes for the normalization of RT-qPCR in different sample sets of *O*. *fragrans* using geNorm, NormFinder and BestKeeper. For RT-qPCR using SYBR Green, *OfRAN1* and *OfUBC2* were the optimal reference genes for all samples and different cultivars, *OfACT* and *OfEF1α* were the optimal reference genes for different floral developmental stages, and *OfACT* was the optimal reference gene for different temperature treatments. The geometric mean values of the optimal reference gene pairs are recommended to be used for all samples, different cultivars and different floral developmental stages. For RT-qPCR using EvaGreen, *OfUBC2* was the optimal reference gene for all samples and different cultivars, and *OfACT* was the optimal reference gene for different floral developmental stages and different temperature treatments. The use of *Of18S* as a reference gene should be avoided in *O*. *fragrans*. To our knowledge, our study is the first systematic characterization of the expressional stability of reference genes in *O*. *fragrans*.
